# Correlation Between Respiratory Accessory Muscles and Diaphragm Pillars MRI and Pulmonary Function Test in Late-Onset Pompe Disease Patients

**DOI:** 10.3389/fneur.2021.621257

**Published:** 2021-03-01

**Authors:** David Reyes-Leiva, Jorge Alonso-Pérez, Mercedes Mayos, Claudia Nuñez-Peralta, Jaume Llauger, Izaskun Belmonte, Irene Pedrosa-Hernández, Sonia Segovia, Jordi Díaz-Manera

**Affiliations:** ^1^Neuromuscular Disorders Unit, Neurology Department, Hospital de la Santa Creu i Sant Pau, Barcelona, Spain; ^2^Centro de Investigación Biomédica en Red en Enfermedades Raras, Madrid, Spain; ^3^Pneumology Department, Hospital de la Santa Creu i Sant Pau, Barcelona, Spain; ^4^Radiology Department, Hospital de la Santa Creu i Sant Pau, Barcelona, Spain; ^5^Rehabilitation and Physiotherapy Department, Hospital de la Santa Creu i Sant Pau, Universitat Autònoma de Barcelona, Barcelona, Spain; ^6^John Walton Muscular Dystrophy Research Center, Newcastle University, Newcastle, United Kingdom

**Keywords:** Pompe disease, glycogen storage disease type II, MRI, muscular MRI, respiratory insufficiency, accessory respiratory muscles, diaphragm pillars

## Abstract

**Objectives:** Pompe disease is a rare genetic disease produced by mutations in the GAA gene leading to progressive skeletal and respiratory muscle weakness. T1-weighted magnetic resonance imaging is useful to identify fatty replacement in skeletal muscles of late-onset Pompe disease (LOPD) patients. Previous studies have shown that replacement by fat correlates with worse results of muscle function tests. Our aim was to investigate if fat replacement of muscles involved in the ventilation process correlated with results of the spirometry and predicted respiratory muscle impairment in LOPD patients over time.

**Materials and Methods:** We studied a cohort of 36 LOPD patients followed up annually in our center for a period of 4 years. We quantified muscle fat replacement using Mercuri score of the thoracic paraspinal and abdominal muscles and the pillars of the diaphragm. We correlated the combined Mercuri scores of these areas with spirometry results and the need of respiratory support.

**Results:** We found a statistically significant correlation (Spearman test, *p* < 0.05; coefficient of correlation > 0.6) between forced vital capacity seated and lying and fat fraction score of all muscle groups studied. The group of patients who needed respiratory support had higher fat fraction scores than patients not requiring ventilatory support. Higher fat replacement in these areas correlated with worse progression in spirometry values over time.

**Conclusions:** Fat replacement of paraspinal, abdominal, and trunk muscles correlates with results of spirometry and is able to predict worsening in respiratory muscle function tests that could lead to an emerging ventilatory dysfunction. Therefore, the identification of fat replacement in these muscle groups should lead to a closer monitorization of patients. Radiologic evaluation of diaphragm pillars in T1-weighted imaging axial sequences could also be helpful to predict respiratory insufficiency.

## Introduction

Pompe disease or glycogen storage disease type II is a rare genetic disease produced by mutations in the *GAA* gene encoding the enzyme acid alpha glucosidase (AAG). This enzyme catalyzes glycogen into glucose inside the lysosomes. Mutations in the *GAA* gene lead to accumulation of lysosomes loaded with glycogen and autophagic vacuoles inside cells in several tissues but especially in skeletal, smooth, and heart muscles (1). This accumulation triggers only partially known intracellular molecular pathways leading to necrosis and substitution of muscle fibers by fat and fibrous tissue that eventually produces permanent skeletal and respiratory muscle weakness.

Pompe disease is differentiated in two main phenotypes. In infantile-onset Pompe disease, symptoms start during the first months of life and are characterized by hypotonia, general muscle weakness, respiratory impairment, and hypertrophic cardiomyopathy. This phenotype is very severe with an ominous prognosis and is associated with a very low or absent expression of AAG (2). In contrast, in late-onset Pompe disease (LOPD), symptoms start in any moment after 1 year old, being characteristic a slowly progressive weakness affecting proximal muscles of the limbs and/or axial muscles associated or not with respiratory muscle involvement and elevated creatine kinase (CK) values in blood tests in most patients (2, 3). The clinical spectrum in LOPD is heterogeneous, ranging from isolated asymptomatic hyperCKemia to severe muscle weakness and respiratory insufficiency requiring invasive ventilatory support. Enzymatic replacement therapy (ERT) with alglucosidase alfa (Myozyme/Lumizyme®, Sanofi-Genzyme, Cambridge, MA, USA) is the only approved treatment for this disease (4). A single placebo-controlled clinical trials and several open-label studies have already shown the efficacy of ERT maintaining muscle and respiratory function over time (5–8).

Respiratory impairment is a common manifestation of LOPD. Approximately 60–80% of LOPD patients develop respiratory insufficiency during their lifetime (9). Ventilation is a complex process that involves several muscles of the trunk, pharynx, and larynx to accomplish an adequate inspiration and expiration cycle. Diaphragm and intercostal muscles are considered primary respiratory muscles playing a key role expanding the thoracic cavity during inspiration. Other muscles such as scalenus, serratus, sternocleidomastoid, and pectoralis support inspiration when diaphragm and intercostals are weak enough to not achieve an appropriate inspiration (10, 11). Respiratory insufficiency in LOPD patients is mainly produced by diaphragm dysfunction that should be checked routinely with pulmonary function test (PFT) using spirometry. A difference of 10% or more in forced vital capacity (FVC) in sitting position compared to lying is considered a sign of diaphragm muscle weakness (12). Other parameters assessed with conventional spirometry include maximal inspiratory pressure (MIP) that also correlates with diaphragm performance. However, PFTs are often influenced by patient's motivation, and to obtain a perfect test is not always easy from a technical point of view.

In recent years, muscle magnetic resonance imaging (MRI) using T1-weighted imaging (T1w) has proved to be useful identifying skeletal muscle fat replacement in several neuromuscular diseases, including LOPD. Previous studies have shown that trunk and lower limbs muscles are commonly replaced by fat in LOPD patients and that the amount of fat present in these muscles correlates with the degree of muscle impairment measured using different muscle function tests (13, 14). Therefore, muscle MRI could be considered a good biomarker of muscle function in this disease useful to distinguish between mild and severe patients (15). Based on these previous results, we wondered if fat replacement of the muscles involved in the ventilation process correlated with spirometry values or predicted respiratory muscle impairment in LOPD patients over time.

## Materials and Methods

### Description of the Study Population and PFT

This study was a prospective longitudinal cohort study involving 36 genetically confirmed LOPD patients performed at Hospital de la Santa Creu i Sant Pau (HSCP) in Barcelona from December 2013 to June 2018. The clinical and genetic features of this cohort have been previously described (15). All patients were seen once per year, and the assessment included a clinical interview, several muscle function tests, a conventional spirometry, and a whole-body muscle MRI using T1w sequence. We obtained PFT during the 4 years' duration of the study, and MRI T1w whole body during the first 3 years. On the fourth year, we changed the MRI protocol, and only images of the lower limbs were obtained, so we did not use those images in the present study. The study was approved by the HSCP Ethics Committee, and all participants signed a consent form. The study was registered in ClinicalTrials.gov with the identifier NCT01914536.

Inclusion criteria for the study were (1) a diagnosis of LOPD according to the recommendations made by the European Pompe Consortium: reduced enzymatic activity on fibroblasts, blood leukocytes or skeletal muscle and/or the presence of two known mutations in the GAA gene (16); (2) no contraindications for the MRI; and (3) willingness to complete muscle function test and respiratory assessment. Asymptomatic patients who only had isolated hyperCKemia without clear skeletal muscle weakness in clinical examination or respiratory symptoms were also included. The criteria requested by health authorities to approve ERT in Spain vary from one region to another, but are commonly based on the presence of skeletal and/or respiratory muscular weakness in clinical examination. Isolated hyperCKemia associated or not to fatigue or muscle pain is not considered a criterion to start ERT.

Muscle function tests and spirometry were performed by two physiotherapists with a long experience in the assessment of neuromuscular patients (I.B., I.P.-H.). In terms of spirometry, we measured FVC seated and lying and MIP and maximal expiratory pressure (MEP) with the Carefusion Microlab ML 3500 MK8 spirometer (Carefusion, Yorba Linda, CA, USA).

### Muscle Imaging

The 36 LOPD patients were imaged in a 1.5-T field magnetic resonance system (1.5-T Achieva, Philips, Eindhoven, the Netherlands) at HSCSP in Barcelona during the whole duration of the study. Patients were imaged in supine position with the legs stretched out. Whole-body axial T1w images were obtained. The images were analyzed by three members of our team (D. R.-L., C. N.-P., J. D.-M.) who quantified fatty muscle replacement using the modified version of the Mercuri score published by Dr. Fischer (17): no fatty replacement: 0 point; mild fatty replacement or traces of infiltration on T1w scores, 1 point; fatty replacement in <50% of the muscle scores, 2 points; fatty replacement in more than 50% of the muscle scores, 3 points; and end-stage appearance with the whole muscle replaced by fatty tissue scores, 4 points.

**Figure 1:**
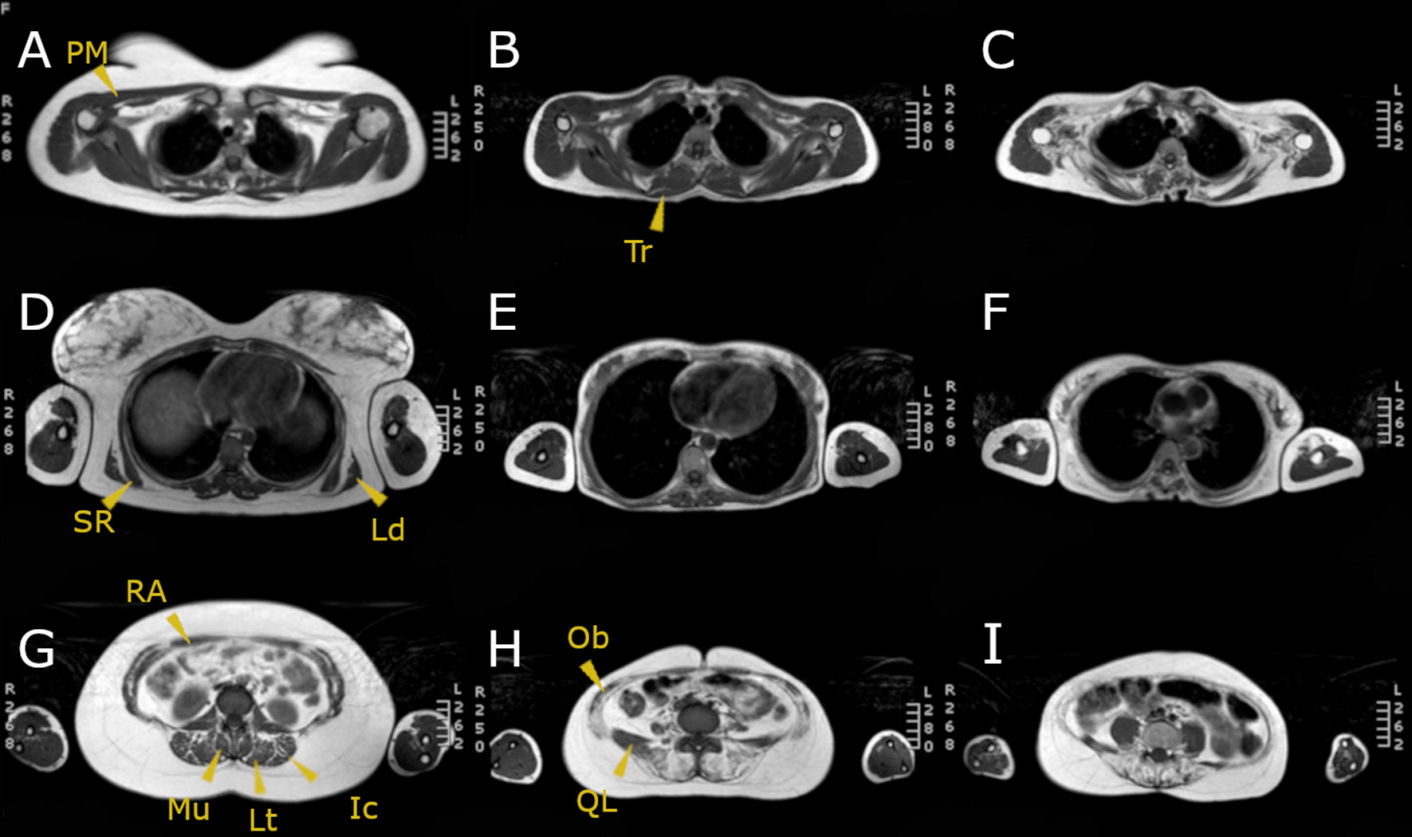
MRI T1w of three LOPD patients: **(A,D,G)** correspond to a patient with mild involvement; **(B,E,H)** correspond to a patient with moderate muscular involvement; **(C,F,I)** correspond to a patient with severe muscular involvement. Yellow arrowheads point the muscles assessed. PM, pectoralis major; SR, serratus; Ld, latissimus dorsi; RA, rectus abdominis; Mu, multifidus; Lt, longissimus thoracis; Ic, iliocostalis; Tr, trapezius; Ob, obliquus externus and internus; QL, quadratus lumborum.

We analyzed fat replacement of the so-called respiratory accessory muscles both in the right and left sides of the body as shown in Figure 1. We divided respiratory accessory muscles in three groups: thoracic muscles, paraspinal muscles, and abdominal muscles. We calculated a compound modified Mercuri score of these areas adding the individual Mercuri score for each of those muscles from both sides (left and right) and eventually obtaining four compound scores:

- Thoracic muscle score: Serratus L (0–4) + serratus R (0–4) + latissimus dorsi L (0–4) + latissimus dorsi R (0–4) + pectoralis major L (0–4) + pectoralis major R (0–4) + scalenus L (0–4) + scalenus R (0–4) + trapezius L (0–4) + trapezius R (0–4). This score ranged from 0 to 40.
- Paraspinal muscle score: Multifidus L (0–4) + multifidus R (0–4) + longissimus thoracis L (0–4) + longissimus thoracis R (0–4) + quadratus lumborum L (0–4) + quadratus lumborum R (0–4) + iliocostalis L (0–4) + iliocostalis R (0–4). This score ranged from 0 to 32
- Abdominal muscle score: Obliquus externus L (0–4)+ obliquus externus R (0–4) + obliquus internus L (0–4) + obliquus internus R (0–4) + transversus abdominis L (0–4) + transversus abdominis R (0–4)+ rectus abdominis L (0–4) + rectus abdominis R (0–4). This score ranged from 0 to 32.
- Global Score: Thoracic muscle score (0–40) + paraspinal muscle score (0–32) + Abdominal muscle score (0–32). This last score ranged from 0 to 104.

**Figure 2:**
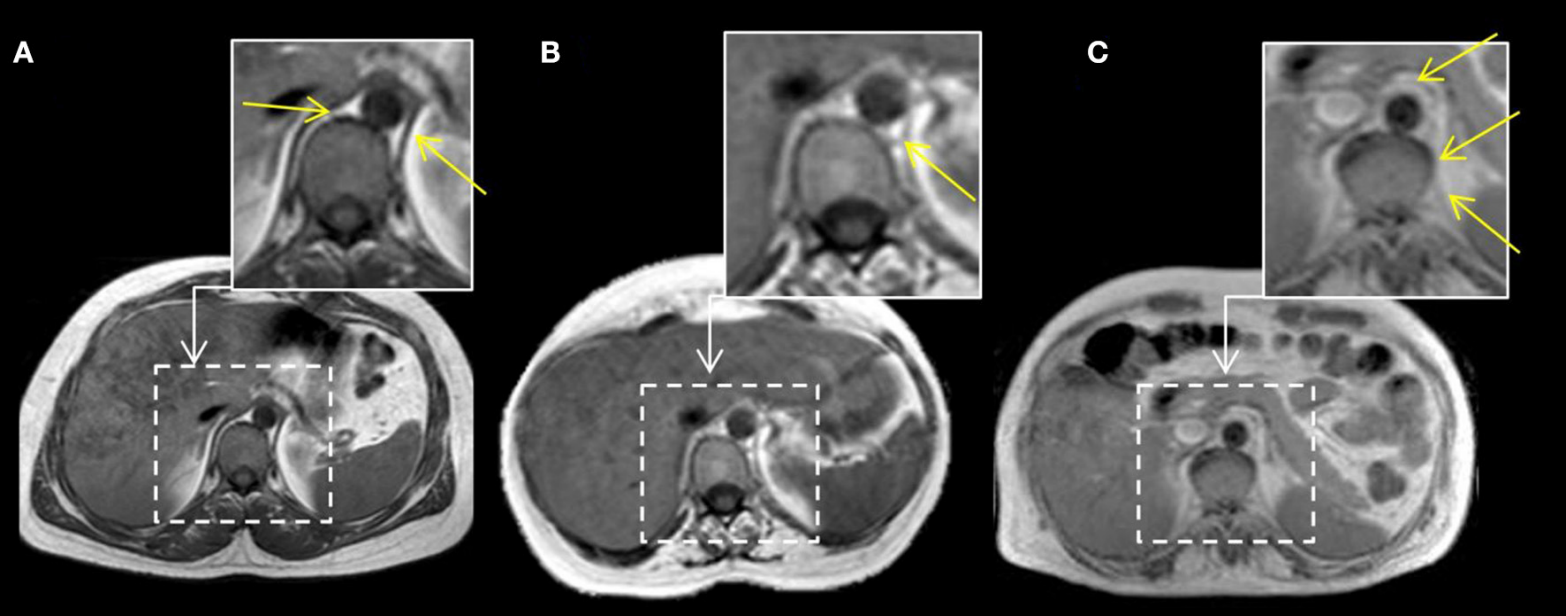
MRI T1w abdominal sections of three LOPD patients. Yellow arrows point to both diaphragm pillars. In case **(A)**, right and left diaphragm pillars are visible and do not show fatty replacement (Mercuri score 0). In case **(B)**, left diaphragm pillar is visible and partially infiltrated, whereas right diaphragm pillar is absent (left pillar Mercuri score 3 points). In case **(C)**, we can observe fully replaced diaphragm pillars at both sides (Mercuri score 4 points).

Furthermore, we assessed the involvement of the diaphragm by analyzing fatty replacement of the diaphragm pillars, which are two musculotendinous structures easily identifiable surrounding the anterior lumbar spine as shown in Figure 2.

### Statistics

We confirmed the scores obtained were normally distributed using Kolmogorov–Smirnov test, and consequently, we used parametric statistic studies to analyze the data. We used Student *t*-test to study if the differences observed in the fatty replacement scores obtained between ventilated and nonventilated patients were significant. To analyze differences observed over time in fatty replacement, we used a mixed linear model and applied Greenhouse–Geisser test. We used Pearson test to correlate respiratory measurements with the degree of muscle involvement, and we considered that the correlation was good if the correlation coefficient were higher than 0.6. Hierarchical analysis and graphical representation as a heatmap was performed using R software, V.3.1.3. Significance was set up at *p*-values lower than 0.05. Statistical studies were performed with SPSS for MAC computers (version 21, SPSS Inc., Chicago, IL).

## Results

### Cohort Description

Clinical data of the 36 LOPD patients enrolled in the study have been previously reported and are displayed in Table 1 (15). Twenty patients were female (55.5%). Mean age of the patients at the start of the study was 43.9 ± 14.8 years. At the baseline visit, 23 patients were on ERT with Myozyme. One patient (patient 7) started ERT during the follow-up. Mean duration of ERT treatment at baseline was 4.30 ± 2.69 years. Eight patients were considered asymptomatic as they only presented hyperCKemia and were not receiving ERT. The most common clinical feature of the cohort was muscular weakness involving the lower limbs. Eleven patients needed noninvasive nocturnal mechanical ventilation at the start of our study. Patient 7 started noninvasive nocturnal mechanical ventilation during the study.

**Table 1:** Clinical features of the cohort.

**Patient**	**Sex**	**Age at diagnosis**	**Age at baseline visit**	**Age at ERT**	**Clinical phenotype**	**Age at ventilation**	**Mechanical ventilation at Baseline Visit**	**Mechanical ventilation at last visit**
1	Female	38	50	47	MW	–	No	No
2	Female	20	48	39	MW + RS	34	Yes (noninvasive)	Yes (noninvasive)
3	Female	–	26	No	HyperCKemia	–	No	No
4	Female	56	63	59	MW	–	No	No
5	Female	20	45	42	MW	–	No	No
6	Female	36	51	47	MW	–	No	No
7	Male	62	66	67	MW + RS	67	No	Yes (noninvasive)
8	Female	49	59	52	MW	–	No	No
9	Female	29	55	48	MW	–	No	No
10	Male	13	42	39	MW + RS	38	Yes (noninvasive)	Yes (noninvasive)
11	Female	23	31	24	MW + RS	23	Yes (noninvasive)	Yes (noninvasive)
12	Female	20	46	39	MW	–	No	No
13	Male	43	47	45	MW + RS	44	Yes (noninvasive)	Yes (noninvasive)
14	Male	41	51	45	MW + RS	50	Yes (noninvasive)	Yes (noninvasive)
15	Female	35	51	46	MW + RS	46	Yes (noninvasive)	Yes (noninvasive)
16	Male	–	22	No	HyperCKemia	–	No	No
17	Male	–	14	No	HyperCKemia	–	No	No
18	Female	40	65	64	MW	–	No	No
19	Female	24	35	29	MW	–	No	No
20	Female	29	40	40	MW + RS	39	Yes (noninvasive)	Yes (noninvasive)
21	Female	20	52	45	MW	–	No	No
22	Male	47	64	57	MW + RS	56	Yes (noninvasive)	Yes (noninvasive)
23	Male	2	8	No	HyperCKemia	–	No	No
24	Female	42	57	55	MW + RS	54	Yes (noninvasive)	Yes (noninvasive)
25	Male	41	46	43	MW + RS	43	Yes (noninvasive)	Yes (noninvasive)
26	Male	35	51	51	MW + RS	49	Yes (noninvasive)	Yes (noninvasive)
27	Male	35	51	No	MW	–	No	No
28	Male	20	43	43	MW	–	No	No
29	Female	40	54	48	MW	–	No	No
30	Male	–	12	No	HyperCKemia	–	No	No
31	Male	49	51	No	HyperCKemia + OA.	–	No	No
32	Male	39	43	No	MW	–	No	No
33	Female	16	20	No	HyperCKemia	–	No	No
34	Female	28	39	No	MW	–	No	No
35	Female	35	35	No	HyperCKemia	–	No	No
36	Male	44	49	No	HyperCKemia	–	No	No

*Age is expressed in years. HyperCKemia patients did not have other clinical manifestations except of patient 31. ERT, enzyme replacement therapy; MW, muscular weakness; RS, respiratory symptoms; OA, obstructive apnea*.

### MRI T1w Analysis

**Figure 3:**
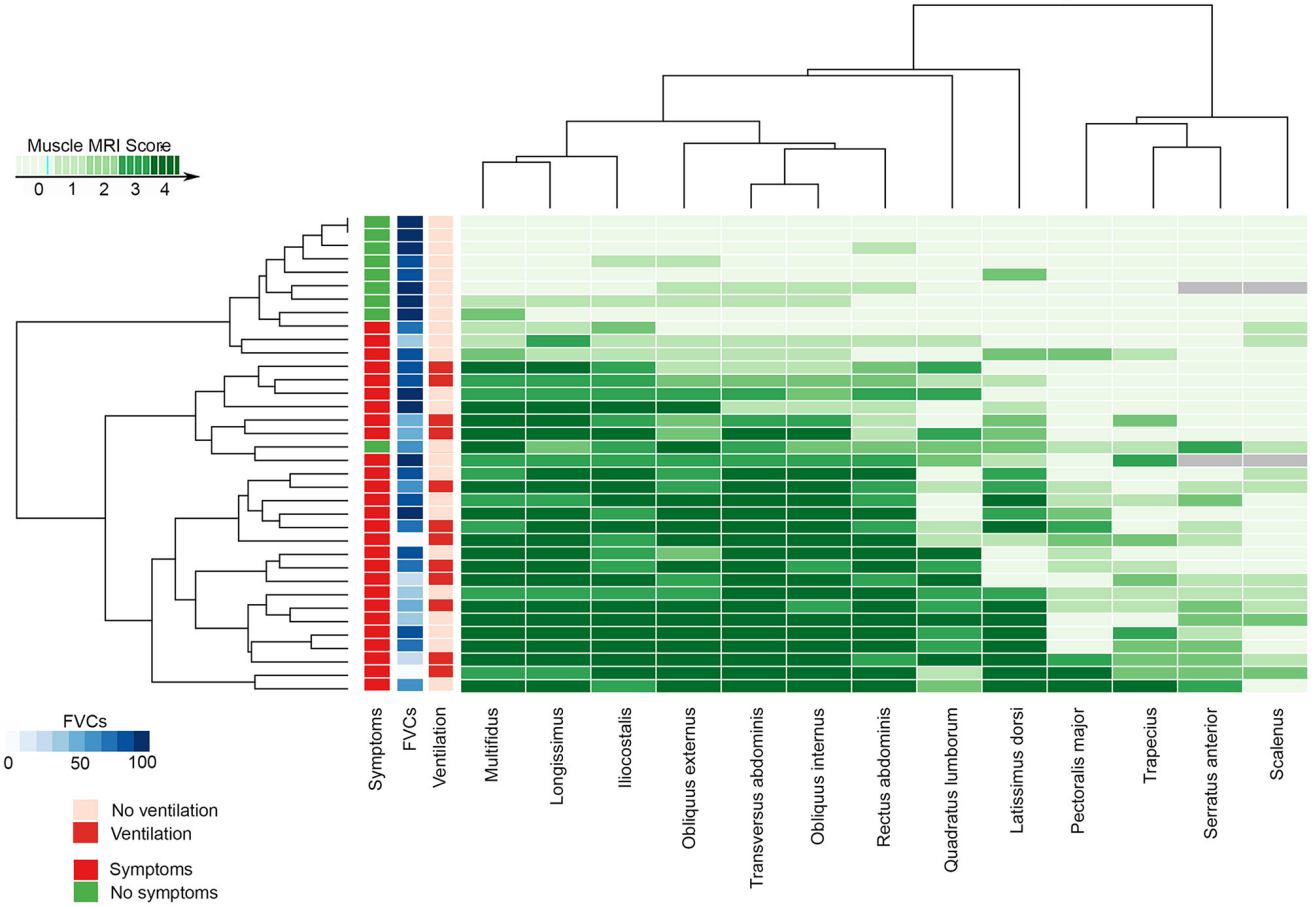
The heatmap shows the MRI Mercuri score value for all the muscles studied, the presence of muscular weakness (symptomatic) or hyperCKemia alone (no symptomatic), its relationship with the FVC at base time, and the needs of noninvasive mechanical ventilation in every patient. FVC, forced vital capacity; MRI, magnetic resonance imaging.

Both abdominal and paraspinal muscles were more commonly and severely affected than thoracic muscles in our cohort as shown in Tables 2, 3 and in Figure 3. The muscles more commonly replaced by fat were multifidus, longissimus, iliocostalis, rectus abdominis, the oblique muscles and transversus abdominis. Latissimus dorsi was the thoracic muscle more common and severely affected, while there was a clear heterogeneity in the involvement of the remaining thoracic muscles among patients as shown in Figure 3. We observed significant differences in the degree of muscle fat replacement between ventilated and nonventilated patients in abdominal and paraspinal muscles but not in thoracic muscles at baseline (Table 3). It is noteworthy that the pillars of the diaphragm were more severely affected in ventilated than in nonventilated patients (Table 3) as shown in Figure 2. There was a significant progressive increase in the degree of fat replacement of thoracic, abdominal, and paraspinal muscles during the follow-up (Greenhouse Geisser, *p* < 0.001). Interestingly, some of the presymptomatic patients have mild signs of fatty replacement especially in paraspinal and abdominal muscles. It is noteworthy that one of the considered presymptomatic patients had more consistent changes affecting also thoracic muscles. However, this patient did not complain of any muscular symptom.

**Table 2:** Mean values of the compound MRI scores at every visit.

	**Visit 1, mean (SD)**	**Visit 2, mean (SD)**	**Visit 3, mean (SD)**
Abdominal score	19.52 (12.5)	19.77 (12.5)	21.5 (11.86)
Paraspinal score	19.11 (11.17)	20.08 (11.4)	21.78 (10.77)
Thorax score	9.08 (9.57)	9.97 (9.53)	11.55 (10.16)
Global score	48.32 (29.78)	49.83 (30.58)	54.89 (29.60)

*SD, standard deviation*.

**Table 3:** Mean values of the MRI scores and Mercuri score of diaphragm pillars classified in ventilated and nonventilated patients.

	**Noninvasive mechanical ventilation = 12 (SD)**	**Nonventilated patients = 24 (SD)**	***p*-value**
Abdominal score	26.16 (6.8)	16.95 (13.62)	0.013
Paraspinal score	26.8 (3.68)	15.54 (11.94)	0.001
Thorax score	12.7 (9.16)	7.7 (9.64)	0.155
Global score	65.75 (14.32)	40.6 (32.53)	0.006
Diaphragm pillar left	2.66 (1.7)	1.2 (1.6)	0.036
Diaphragm pillar right	2.66 (1.7)	1.2 (1.6)	0.036

*The last column shows results of Student t-test (significant p < 0.05). SD = standard deviation*.

### Pulmonary Function Test Analysis

There were significant differences in FVC percentage predicted measured seated or lying between ventilated and nonventilated patients (Student *t*-test, *p* = 0.001 for both measurements). Moreover, we identified a significant difference in FVC seated between baseline and last visit both in ventilated and nonventilated patients (Table 4). We did not observe significant differences in FVC in presymptomatic patients between baseline and last visit. The MIP and MEP values at baseline visit were also studied obtaining a mean MIP of 70.52 ± 28.95% and a mean MEP 72.98 ± 31.77% in all the patients.

**Table 4:** Mean values of the FVC measured seated and mean values of the differences between FVC seated and lying at the first and last visit.

	**First visit, mean FVC% seated (SD)**	**Last visit, mean FVC% seated (SD)**	***p*-value**
All patients (36)	79.98% (21.43)	73.42 % (25.34)	0.002
Nonventilated patients (24)	88.23% (18.61)	80.94% (24.21)	0.017
Ventilated patients (12)	65.39% (12.91)	59.7% (14.06)	0.018
	**First visit, mean FVC% S-L (SD)**	**Last visit, mean FVC% S-L (SD)**	***p*****-value**
All patients (36)	11.51 (12.27)	8.46 (7.42)	0.107
Nonventilated patients (24)	10.45 (11.48)	8.11 (6.71)	0.460
Ventilated patients (12)	23.16 (15.00)	11.00 (8.93)	0.057

*We compared the mean values from the first and last visit (p-values in the last column). FVC%, forced vital capacity percentage predicted; FVC% S-L, FVC% measured seated – FVC% measured lying; SD, standard deviation*.

### Correlation Test

We observed significant correlation between global, thoracic, abdominal, and paraspinal scores and FVC percentage predicted seated and lying at baseline. Additionally, there were significant correlations between fat replacement of the diaphragm pillars and FVC percentage predicted seated and lying and MIP (Table 5).

**Table 5:** Correlations found between the compound scores calculated and the diaphragm pillar involvement and the FVC% seated and lying.

	**FVC% seated**	**FVC% lying**	**MIP**	**MEP**
Global score	*p* = 0.002 *R* = −0.52	*p* = 0.004 *R* = −0.608	*p* = 0.772	*p* = 0.135
Thorax score	*p* = 0.001 *R* = −0.541	*p* = 0.003 *R* = −0.621	*p* = 0.372	*p* = 0.124
Abdominal score	*p* = 0.011 *R* = −0.523	*p* = 0.021 *R* = −0.394	*p* = 0.301	*p* = 0.248
Paraspinal score	*p* = 0.007 *R* = −0.456	*p* = 0.007 *R* = −0.570	*p* = 0.308	*p* = 0.309
Diaphragm pillars	*p* = 0.0001 *R* = −0.590	*p* = 0.001 *R* = −0.662	*p* = 0.006 *R* = −0.689	*p* = 0.59

*We are showing the results of the Pearson test: p-value and R value. FVC%, forced vital capacity percentage predicted; MIP, maximal inspiratory pressure; MEP, maximal expiratory pressure*.

We also observed a significant correlation between baseline MRI score and changes in FVC percentage predicted after 3 years of follow-up for global (*p* = 0.013, *r* = −0.506), abdominal *p* = 0.017, *r* = −0.602), and paraspinal (*p* = 0.013, *r* = −0.542) MRI Score. Although significant, the correlations with thoracic MRI score and fat replacement of the diaphragm pillar were low: *r* = −0.233 and *r* = −0.437, respectively.

It is noteworthy that one of the patients ventilated (patient 24), with a clear diaphragmatic dysfunction characterized by a drop in FVC when lying higher than 20%, had mild abdominal but severe paraspinal fat replacement (abdominal score: 10, paraspinal score: 28) and normal diaphragm pillars. Additionally, we detected patients (patients 6, 9, 14, 29, and 31) who had mild to moderate abdominal fat replacement with a severe paraspinal fat replacement but with diaphragm pillars nonaffected and who did not show diaphragm dysfunction (FVC Seated - FVC lying <10%).

**Figure 4:**
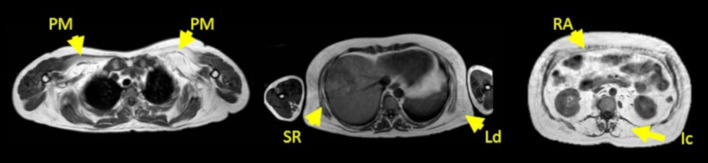
MRI T1w of patient 14 at first visit. From left to right, first image corresponds to shoulder girdle level; second image corresponds to a thoracic level, and third image corresponds to an abdominal level. Yellow arrows point to the pectoralis major (PM), serratus (SR), latissimus dorsi (Ld), rectus abdominis (RA), and iliocostalis muscle (Ic). The MRI scores at this visit were 30 points in abdominal score, 25 points in paraspinal score, and 18 points in thoracic score. The Global score was of 73 points.

We want to highlight the individual case of patient 14 who is a 51-year-old male patient who was already using nocturnal ventilation when recruited in this study. The patient was diagnosed as having sleep apnea 3 years before being recruited and was on nocturnal ventilation 8 h per night at the beginning of the study, and this remained unmodified the whole duration of the study. The predicted FVC seated was 78%, and the predicted FVC lying was 83% at baseline. At last visit, predicted FVC seated was 69%, and FVC lying was 64%. These differences were not compatible with diaphragmatic dysfunction using the definition mentioned above (a drop in FVC higher than 10% between seating and lying), but a worsening tendency was noticed. In this patient, muscle MRI showed a severe fatty replacement of paraspinal and abdominal muscles that was not modified throughout the whole duration of the study. We have included an MRI of this patient at first visit in Figure 4. The MRI scores measured for this patient on the baseline visit were 30 points in abdominal score, 25 points in paraspinal score, and 18 points in thoracic score, so the Global score at the first visit was of 73 points. In the last visit, we identified a very mild worsening in paraspinal score (26 points) and in thoracic score (19 points) remaining unchanged the abdominal score. The global score was of 75 then.

## Discussion

In this study, we show that fat replacement of thoracic, abdominal, and paraspinal muscles correlates with low values of FVC percentage predicted both seated and lying in a large cohort of LOPD patients. Additionally, fat replacement of abdominal and paraspinal muscles predicted changes in FVC during the follow-up in our cohort. These results suggest that identification of fat replacement in these areas on the MRI should lead to a closer monitorization of respiratory muscle function over time.

It is well known that LOPD patients have a selective involvement of paraspinal, abdominal and proximal muscles of the lower limbs in T1w MRI sequences (14, 15). Here we have confirmed these previous results, but we have also found a good correlation between fat replacement and results of respiratory muscle function tests. It has been previously shown that the degree of fat replacement in a muscle correlates with results of specific function tests including assessment of muscle strength using handheld dynamometry or even assessment of muscle performance using timed tests such as the 6-min walking test (18–20). Accordingly, it is not surprising that fat replacement of the diaphragm or of the accessory respiratory muscles, such as thoracic muscles, has a good correlation with FVC. However, we have seen here that abdominal and paraspinal involvement is also associated with FVC, and it predicts changes in this measure over time. This also suggests that clinical examination findings such as a distended abdominal wall, Beevor sign (cephalic umbilical shift with abdominal muscular contraction due to abdominal weakness), or axial weakness, which are a common finding in LOPD, should also point to a closer monitorization of respiratory function (21).

In concordance with these results, we have observed significant differences in the degree of fat replacement between ventilated and nonventilated LOPD patients, suggesting that fat replacement of these muscles leads to a decrease in respiratory muscle function and eventually to the need of nocturnal respiratory support. All patients using noninvasive ventilation had some degree of fatty replacement in diaphragm pillars, except patient 14, who despite being ventilated did not meet the standard criteria of diaphragm dysfunction. Although diaphragm pillar grading is not a standardized procedure, and there is a wide variability between healthy volunteers and patients, we consider it is a valid measure linked to clinical involvement of the diaphragm.

We have identified significant differences in FVC between baseline and last visit in symptomatic patients treated despite being treated with ERT. Several other studies have also analyzed the progression of FVC over time in LODP patients treated with ERT, and the results are variable. Most of the studies coincide in a positive effect of starting ERT on FVC, as this value remains stable in most of the patients during the first 5 years of treatment (5, 8, 22). However, a recent study including patients followed up for 10 years identified a mean decrease of more than 10% in FVC in patients treated, which is in agreement with our results, which are supported by an increase in fat replacement of respiratory muscles, suggesting that ERT is not able to completely stop progression of respiratory muscle degeneration in Pompe patients (7).

Other previous studies have identified a correlation between fatty replacement of abdominal muscles and diaphragm using computed tomography scan or MRI with spirometry results (23). We have confirmed these previous results, and additionally, we have also found a correlation with fatty replacement of paraspinal and thoracic muscles such as scalenus, trapezius, and pectoralis major. However, it is noteworthy that the correlation between FVC and fatty replacement of thoracic muscles was low, suggesting that they do not have a prominent role in respiratory function in LOPD patients, which is probably more dependent of the diaphragm and the abdominal muscles in these patients. Our protocol of MRI included only axial images, which unfortunately do not allow a good analysis of intercostalis muscles. We think that coronal images should be obtained if these muscles want to be analyzed. In recent years, several studies have also used MRI to study diaphragm function, and they have elegantly shown changes in morphology of thorax cavity and decrease in thorax dynamics during inspiration and expiration in LOPD patients (23–25). These results suggest a prominent involvement of the diaphragm, which is also supported by the frequent finding of a decrease in FVC percentage predicted higher than 10% when patients are lying compared to FVC seating and by the results of echography studies showing thinner diaphragms, which are replaced by fat (11, 26). The biggest limitation of our study is that we have used a semiquantitative scale to analyze fat replacement that is observer dependent and that have several limitations to identify consistent changes over time. At present, quantitative muscle MRI techniques, such as Dixon, are available and have demonstrated to identify better changes in fat replacement over time (27–29). However, it is important to take into account that Dixon studies of the trunk muscles are extremely challenging because of the movement artifacts of this area during normal respiration. To obtain images of good quality could be even more difficult in patients with respiratory involvement and orthopnea such as LOPD patients because the only way of avoiding motion artifacts using Dixon in this body areas is asking the patients to do repeated apneas that can last for 10–15 s (15, 30).

In conclusion, fat replacement of paraspinal, abdominal, and trunk muscles correlates with FVC percentage predicted seated and lying and is able to predict worsening in respiratory muscle function tests that could lead to an emerging ventilatory dysfunction. Therefore, the identification of fat replacement in these muscle groups should lead to a closer monitorization of patients.

## Data Availability Statement

The raw data supporting the conclusions of this article will be made available by the authors, without undue reservation.

## Ethics Statement

The studies involving human participants were reviewed and approved by Ethics committee from Institut de Recerca Biomédica Hospital de la Santa Creu i Sant Pau. The patients/participants provided their written informed consent to participate in this study.

## Author Contributions

DR-L, JA-P, CN-P, MM, JL, SS, and JD-M carried out the concept and design of the study. DR-L, JA-P, CN-P, JL, and JD-M performed the acquisition and analysis of data. JD-M performed the statistical analysis. DR-L, JA-P, IB, IP-H, and MM performed the acquisition and analysis of the PFT. DR-L, JA-P, CN-P, JL, SS, MM, and JD-M drafted of the manuscript. All authors contributed to the article and approved the submitted version.

## Conflict of Interest

The authors declare that the research was conducted in the absence of any commercial or financial relationships that could be construed as a potential conflict of interest.
